# Absence of phonon softening across a charge density wave transition due to quantum fluctuations

**DOI:** 10.1073/pnas.2507135122

**Published:** 2025-08-01

**Authors:** Yubi Chen, Terawit Kongruengkit, Andrea Capa Salinas, Runqing Yang, Yujie Quan, Fanghao Zhang, Ganesh Pokharel, Linus Kautzsch, Stephen D. Wilson, Sai Mu, John W. Harter, Bolin Liao

**Affiliations:** ^a^Department of Physics, University of California, Santa Barbara, CA 93106-9530; ^b^Department of Mechanical Engineering, University of California, Santa Barbara, CA 93106-5070; ^c^Materials Department, University of California, Santa Barbara, CA 93106-5050; ^d^Department of Physics, Perry College of Mathematics, Computing, and Sciences, University of West Georgia, Carrollton, GA 30118; ^e^Department of Physics and Astronomy, SmartState Center for Experimental Nanoscale Physics, University of South Carolina, Columbia, SC 29208

**Keywords:** charge density wave, topological kagome metal, quantum zero-point motion, electron–phonon coupling

## Abstract

Kagome metals have emerged as a frontier in condensed matter physics due to their potential to host exotic quantum states. Among these, CsV_3_Sb_5_ has attracted significant attention for the unusual coexistence of charge density wave (CDW) order and unconventional superconductivity, presenting an ideal system for exploring the emergent phenomena from the interplay of phonons, electronic fluctuations, and topological effects. The nature of CDW formation in CsV_3_Sb_5_ is unconventional and has sparked considerable debate. In this study, we examine the origin of the CDW state via ab initio finite-temperature simulations of the lattice dynamics. Through a comparative study of CsV_3_Sb_5_ and 2H-NbSe_2_, we demonstrate that the experimental absence of phonon softening—a hallmark of conventional CDW transition—in CsV_3_Sb_5_ along with the presence of a weakly first-order transition, can be attributed to quantum zero-point atomic motion. This zero-point motion smears the free energy landscape of CDW, effectively stabilizing the pristine structure even below the CDW transition temperature. We argue that this surprising behavior could cause coexistence of pristine and CDW structures across the transition and lead to a weak first-order transition. Our predicted lattice dynamical behavior is supported by coherent phonon spectroscopy in single-crystalline CsV_3_Sb_5_. Our results provide crucial insights into the formation mechanism of CDW materials that exhibit little to no phonon softening, including cuprates, and highlight the surprising role of quantum effects in emergent properties of relatively heavy-element materials like CsV_3_Sb_5_.

## Significance

The charge density wave (CDW) transition is an important phenomenon in condensed matter physics. Typical CDW transitions are driven by strong electron–phonon coupling signaled by phonon softening and lattice instability near the transition temperature. Combining first-principles simulation and experiment, here we challenge this notion by pointing out that quantum zero-point motion can potentially suppress phonon softening across the CDW transition, as recently observed in topological kagome metal CsV_3_Sb_5_ and some high-temperature superconducting cuprates. Using CsV_3_Sb_5_ as a model system, we use finite-temperature lattice dynamics and coherent phonon spectroscopy to probe the free-energy landscape near its CDW transition and point out the surprisingly important role of quantum fluctuations. Our result leads to profound understanding of CDW transitions.

Kagome materials have recently become a focal point in condensed matter physics due to their inherent features such as geometric frustration and flat electronic bands (1, 2). These features enable the potential to host a gamut of exotic quantum phenomena (3–6). Among Kagome materials, the newly discovered $Z_{2}$ topological metal CsV_3_Sb_5_ (7–10) has received intensive research interest. One of the intriguing feature of CsV_3_Sb_5_ is the coexistence of a charge density wave (CDW) order below 94 K and superconductivity below 2.5 K out of a CDW background (11–14). Understanding the nature of its CDW transition is not only crucial for determining pairing mechanisms for the unconventional superconductivity (15, 16) but also important for developing next-generation quantum devices (17). Nevertheless, the precise mechanism driving the CDW order in CsV_3_Sb_5_ remains poorly understood, presenting a substantial challenge both theoretically and experimentally.

Previous research has suggested that conventional factors driving CDW transitions, such as Fermi surface nesting and strong electron–phonon coupling (EPC) may not play a major role in CsV_3_Sb_5_ (18–21). For example, calculations of the Lindhard susceptibility (22–24) have shown that Fermi surface nesting is not the predominant cause due to inconsistent nesting wavevector and the CDW wavevector. In addition, CsV_3_Sb_5_ differs notably from conventional EPC-driven CDW materials [e.g. 2H-NbSe2 (20)] due to the absence of phonon softening across the CDW transition—a hallmark of EPC-induced CDW transitions (20, 21). The absence of phonon softening is supported by Raman and X-ray measurements (25, 26), despite both measurements and simulations indicating moderate EPC strengths (24, 27–30). Explanations for the absence of phonon softening have been suggested, such as a very broad linewidth of the soft phonon mode (31) and a persistent phason gap (32), but the confirmation of these conjectures remains elusive. Additionally, alternative mechanisms for CDW formation that go beyond conventional EPC have been proposed, such as a Jahn–Teller-like transition (23), exciton condensation (33, 34) etc. These approaches have not fully accounted for all the experimental observations, indicating that additional factors might be involved.

Conventional density functional theory (DFT) calculations—the workhorse of modern predictive materials modeling—have been employed to investigate the electronic and phononic properties of CsV_3_Sb_5_ to understand its CDW formation (23, 32). However, these calculations are typically restricted to zero temperature, while the finite-temperature lattice dynamics and quantum zero-point fluctuations are usually overlooked. In this study, we employ DFT to analyze the free energy landscape of CsV_3_Sb_5_ with various CDW distortions. To further account for the effect of thermal and quantum zero-point fluctuations (35), we apply a finite-temperature lattice dynamics technique based on the stochastic self-consistent harmonic approximation (SSCHA) (36) to examine how the free energy landscape and the phonon dynamics impact the CDW transition in CsV_3_Sb_5_.

Through a detailed comparative study with 2H-NbSe2, we elucidate the important and unique role of quantum zero-point motion in suppressing the phonon softening behavior in CsV_3_Sb_5_, stabilizing the pristine structure of CsV_3_Sb_5_ and leading to a weak first-order CDW transition and the coexistence of CDW and pristine phases around the transition temperature. This scenario naturally explains the phonon linewidth broadening observed in our coherent phonon spectroscopy (CPS) measurements on single-crystalline CsV_3_Sb_5_. Our result challenges the prevailing understanding of phonon softening behavior as a necessary indicator of EPC-induced CDW transitions. This work reconciles the possibility of EPC as the origin of CDW, despite the absence of phonon softening in CsV_3_Sb_5_, and provides insights that may be applicable to other unconventional CDW materials like cuprates (20, 21). Moreover, we highlight the surprising significance of quantum zero-point motion in a relatively heavy-element material like CsV_3_Sb_5_. This behavior is related to quantum paraelectricity in SrTiO_3_ (37, 38). However, there are important differences between these two scenarios: Whereas the ferroelectric transition is suppressed by quantum fluctuations in SrTiO_3_, there is a weak first-order CDW transition in CsV_3_Sb_5_. We elaborate on this difference in Section 2.B.

## 1. Methods

### 1.1. Computational Methods.

First-principles DFT calculations were conducted using the Vienna Ab initio Simulation Package version 6.4.1 (39, 40), using the projector augmented wave pseudopotentials (41). A 300 eV plane-wave cutoff was employed for CsV_3_Sb_5_, and 400 eV cutoff for 2H-NbSe2. The exchange-correlation functional was chosen to be the Perdew–Burke–Ernzerhof (PBE) functional (42) with van der Waals (vdW) D3 correction (43), denoted as PBE+vdW. The electronic configurations for the elemental pseudopotentials were Cs ($5s^{2}6p^{6}6s^{1}$), V ($3s^{2}3p^{6}3d^{3}4s^{2}$), Sb ($5s^{2}5p^{3}$), Nb ($4p^{6}4d^{3}5s^{2}$), and Se ($4s^{2}4p^{4}$). Brillouin zone sampling was performed using an 18×18×12Γ-centered **k** grid for CsV_3_Sb_5_ unit cell, and a 24×24×8Γ-centered **k** grid for 2H-NbSe2 unit cell. The converged lattice parameters were a=b=5.4491 Å, c=9.3056 Å for pristine CsV_3_Sb_5_ and a=b=3.4517 Å, c=12.3909 Å for pristine 2H-NbSe2, in a good agreement to experimental data a=b=5.52 Å, c=9.36 Å for CsV_3_Sb_5_ (8) and a=b=3.4446 Å, c=12.5444 Å for 2H-NbSe2 at 298 K (44). Both systems utilized no spin polarization for the absent local spin moments (7, 8) and a Gaussian smearing of 0.1 eV. The convergence threshold was set as $1\times 10^{-7}$ eV for energy and as 0.005 eV/Å for force. The harmonic phonon calculations were executed using the finite-displacement method in Phonopy (45), with supercell sizes of 3×3×2 for CsV_3_Sb_5_ (162 atoms) and 6×6×1 for 2H-NbSe2 (216 atoms), using Γ-centered supercell **k** grids of 3×3×3 and 2×2×2, respectively. We found that the harmonic phonon dispersion in CsV_3_Sb_5_ was strongly affected by the supercell size, whose convergence needs to be carefully checked [See *SI Appendix*, Fig. S1 for the supercell convergence in CsV_3_Sb_5_ pristine phonon]. Spin–orbit coupling (SOC) was turned off after verifying its negligible effect on phonon dispersions [See *SI Appendix*, Fig. S2 for a comparison of pristine phonon dispersions with PBE+vdW+SOC and PBE+vdW].

The SSCHA technique was employed to explore anharmonic and quantum effects on lattice dynamics at a nonperturbative level (46–48) [See *SI Appendix* for more details]. The SSCHA method variationally minimizes the free energy by optimizing internal coordinates while preserving the symmetry of the system. Gaussian ensembles of lattice distortions surrounding an average structure are sampled to estimate the lattice free energy, which is then minimized by adjusting the average structure and the spread of the Gaussian probability distribution including both thermal and quantum effects. Self-consistency was achieved for each temperature by utilizing 200 configurations per ensemble, ensuring that the error ratio to the free-energy gradient stayed below $1\times 10^{-4}$. Once self-consistency was achieved, phonon dispersions at finite temperatures were calculated using the free energy Hessians (47) evaluated with up to 5,000 configurations. The computational setup of ensemble configurations was aligned with the harmonic phonon calculations, maintaining consistency across our methodology.

### 1.2. Experimental Methods.

For the CPS experiment, optical pump–probe transient reflectivity measurements were performed on a freshly cleaved sample of CsV_3_Sb_5_ mounted in an optical cryostat. A noncollinear optical parametric amplifier generated ∼70 fs signal (800 nm) and idler (1,515 nm) pulses at a 50 kHz repetition rate, with a pump fluence of approximately 100 $\mathrm{mJ}/{\mathrm{cm}}^{2}$. Measurements at different temperatures were performed upon successive warming from a base temperature of 9 K. To isolate the coherent phonon oscillations, an exponential background was subtracted from the transient change in reflectivity, followed by an apodization step, and Fourier transforming to the frequency domain. Further details on the experimental technique can be found in ref. 49.

## 2. Results and Discussions

### 2.1. Conventional Phonon Behavior of EPC-Driven CDW Materials.

**Fig. 1.**
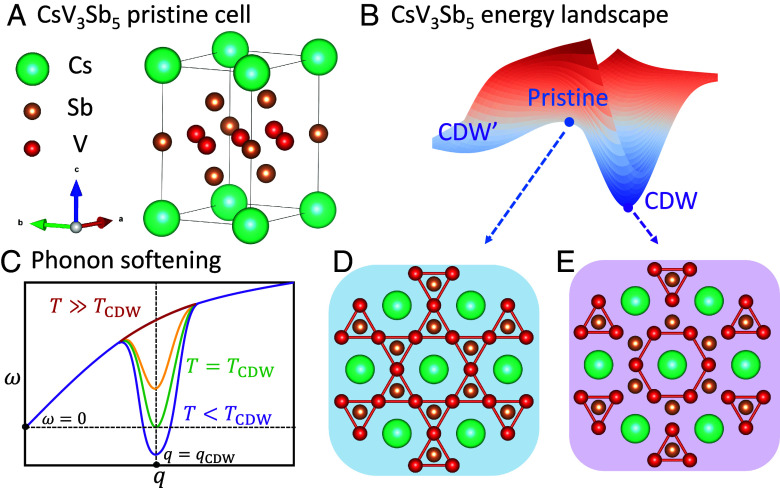
(*A*) Atomic structure of the CsV_3_Sb_5_ pristine cell. (*B*) A schematic of CsV_3_Sb_5_ energy landscape, featuring multiple potential wells representing the CDW structures and a saddle point representing the pristine structure. (*C*) A schematic illustration of typical phonon softening behavior for the pristine structure across the CDW transition temperature $T_{\mathrm{CDW}}$. The phonon mode corresponding to the CDW structure ($q=q_{\mathrm{CDW}}$) is gradually destabilized with a decreasing temperature. Notably, CsV_3_Sb_5_ does not exhibit the phonon softening behavior experimentally. (*D*) *Top*-view of the CsV_3_Sb_5_ pristine structure with symmetry P6/mmm, where V atoms form a Kagome lattice. The V–V pairs are connected with the same bond length 2.725 Å. (*E*) *Top*-view of the CsV_3_Sb_5_ trihexagonal (TrH) layers. The V–V bond distance cutoff is set as 2.7 Åfor visualization, and pairs of V atoms that exceed this distance are not connected.

Fig. 1*A* illustrates the pristine cell of CsV_3_Sb_5_, featuring a layered structure composed of alternating atomic planes. The vanadium (V) atoms are arranged in a two-dimensional Kagome lattice, surrounded by hexagonal antimony (Sb) atoms. The Kagome plane is sandwiched between two honeycomb Sb layers, with cesium (Cs) atoms situated between the V-Sb layers. CDW materials typically transition from a high-symmetry pristine structure to a low-symmetry CDW structure as the temperature decreases. For instance, CsV_3_Sb_5_ transitions from a P6/mmm structure at room temperature, depicted in Fig. 1*D*, to a CDW structure at 94 K, which is the trihexagonal (TrH) 2×2×2 or mixed TrH star-of-David (SoD) 2×2×4 configuration (9, 25, 50) with an in-plane TrH layer shown in Fig. 1*E*. Similarly, 2H-NbSe2 undergoes a transition into its CDW structure at 33 K (51) [See *SI Appendix* for more details about the CDW order in 2H-NbSe2 (52–54)]. From ground-state DFT calculations conducted at zero temperature, the CDW structures are energetically favorable compared to the pristine structure. Considering a continuous variation of structural coordinates, where each structure correlates with one energy, we can visualize an energy landscape, as illustrated in Fig. 1*B*. In this energy landscape, the potential wells correspond to the energetically stable CDW structures, and the saddle point indicates the unstable pristine structure.

EPC-driven CDW transitions are often accompanied by a phonon softening behavior sketched in Fig. 1*C* (51, 55). Due to the unstable saddle point in the energy landscape, the pristine structure at zero temperature should display imaginary phonon frequencies at phonon wavevectors corresponding to the CDW distortions, as depicted by the purple curve in Fig. 1*C*. As the temperature increases, the imaginary phonon is gradually suppressed, which becomes stable (ω≥0) above the CDW transition temperature ($T\geq T_{\mathrm{CDW}}$). For instance, experimental observations of EPC-driven CDW in 2H-NbSe2 show these typical softening behaviors illustrated in Fig. 1*C* (51). In contrast, CsV_3_Sb_5_ does not follow this pattern: Experimental results reveal no phonon softening (25, 26), which was previously interpreted to suggest that EPC is not the dominant mechanism driving the CDW transition in CsV_3_Sb_5_.

**Fig. 2.**
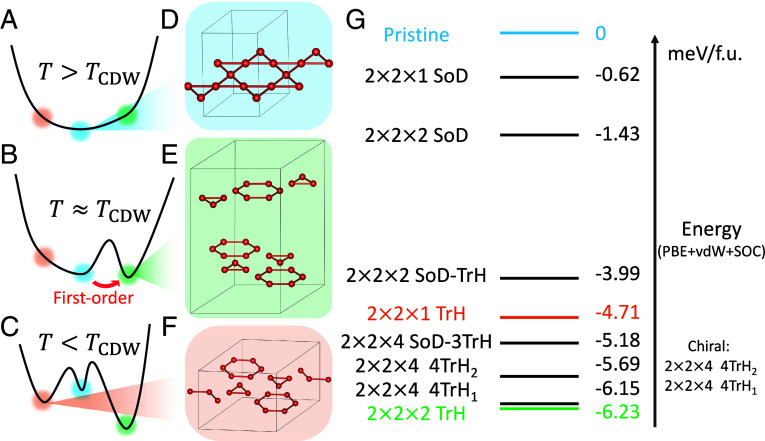
The schematic evolution of the free energy landscape at (*A*) above the CDW transition temperature ($T>T_{\mathrm{CDW}}$), (*B*) near the transition temperature ($T\approx T_{\mathrm{CDW}}$), and (*C*) below the transition temperature ($T<T_{\mathrm{CDW}}$). (*D*) The pristine structure of CsV_3_Sb_5_. (*E*) The CDW order with 2×2×2 TrH layers. (*F*) The CDW order with 2×2×1 TrH layers. (*G*) The energy hierarchy of identified CsV_3_Sb_5_ CDW orders in the units of meV per formula unit (meV/f.u.).

Fig. 2*A*–*C* illustrates the schematic evolution of the free energy landscape in CsV_3_Sb_5_ as the temperature decreases. In Fig. 2*A*, above the CDW transition temperature ($T>T_{\mathrm{CDW}}$), the pristine structure, which is labeled by the blue dot with the atomic structure shown in Fig. 2*D*, represents a global free energy minimum and the system favors the high-symmetry pristine state. As the temperature approaches the CDW transition ($T\approx T_{\mathrm{CDW}}$) in Fig. 2*B*, low-symmetry CDW structures, like the 2×2×2 TrH structure shown in Fig. 2*E* indicated by the green dot, exhibit free energies comparable to that of the pristine structure, facilitating the onset of the CDW transition. Below the transition temperature ($T<T_{\mathrm{CDW}}$) in Fig. 2*C*, additional CDW orders, such as the orange dot 2×2×1 TrH order in Fig. 2*F*, become local minima with lower free energies than the pristine phase. The rationale for depicting the pristine structure as a local minimum at all temperatures will be discussed in the next section.

We systematically explored and identified multiple possible CDW structures in CsV_3_Sb_5_. Fig. 2*G* provides an overview of the energy hierarchy of identified CDW structures as compared to the pristine structure, computed using the PBE+vdW+SOC method. The energies of the structures are directly comparable, as they were calculated using consistent setups, with computational details and their atomic structures provided in *SI Appendix*. Among these, the 2×2×2 TrH order is the most energetically favorable structure with a stable phonon dispersion. The 2×2×4 4TrH_1_ and 4TrH_2_ chiral structures, both consisting of four TrH layers, exhibit energies close to the global minimum, which can potentially explain the chirality observed in experiments (56). The 2×2×4 SoD-3TrH structure, comprising one layer of SoD and three layers of TrH, corresponds to the structure reported in ref. 57. The 2×2×1 TrH structure exhibit a stable phonon dispersion, similar to the global minimum [See *SI Appendix* for the stable phonon dispersions of 2×2×1 and 2×2×2 TrH orders (26, 58, 59)]. The structures with SoD layers are found to be more unstable. The 2×2×1 SoD is dynamically unstable with imaginary phonon frequencies. The 2×2×2 SoD-TrH, composed of one SoD layer and one TrH layer, and 2×2×2 SoD, composed of two SoD layers, only emerge under less stringent convergence criteria, as they tend to relax back to the 2×2×2 TrH configuration under tighter conditions.

Overall, Fig. 2*G* also provides an estimation of the energy barriers between these distinct structures. The energy barrier between the ground state structure and the pristine phase is about 6.23 meV per formula unit (f.u.), which corresponds to 49.84 meV(=6.23×8) for the degree of freedom associated with the distortion into a 2×2×2 CDW structure. Given that the CDW transition occurs at 94 K (≈8 meV), this barrier is unlikely to be overcome solely by thermal fluctuations. In the following section, we will show that the energy barrier could be reduced due to the effects of zero-point motion.

### 2.2. Quantum Zero Point Motion.

To effectively grasp the impact of finite temperatures on CDW materials, it is crucial to incorporate both thermal fluctuations and quantum zero-point effects on their lattice dynamics, for which we employ the SSCHA method (36). Specifically, the SSCHA method aims to variationally minimize the free energy F=E−TS, where E represents the energy and TS denotes the entropy contribution (60). During the minimization process, SSCHA generates a set of ensemble configurations based on statistical fluctuations involving both the thermal and zero-point effects.

Zero-point motion in a lattice arises from the quantum mechanical effect that introduces position uncertainty. The Hamiltonian of the lattice can be transformed to a set of independent harmonic oscillators. For a single harmonic oscillator at zero temperature, the position uncertainty of the ground state is given by $\left\langle x_{i}^{2}\right\rangle =\frac{\hbar }{2m\omega_{i}}$, where m is the effective mass of a particle in a harmonic potential with frequency $\omega_{i}$, and $x_{i}$ is the displacement amplitude relative to the coordinates of the reference structure, which is the pristine structure in our case. The mass-weighted distance from the pristine structure is $\sqrt{\left\langle mx_{i}^{2}\right\rangle }=\sqrt{\frac{\hbar }{2\omega_{i}}}$, which depends solely on the vibrational frequencies. To simulate the quantum effects and thermal effects, we can generate an ensemble of configurations, where position $x_{i}$ is drawn from a Gaussian distribution, determined by the position uncertainty at finite temperatures. By evaluating the energy of the ensemble configurations via DFT, we can extract various ensemble-averaged physical properties, such as the finite-temperature average structure, phonon dispersions, etc.

**Fig. 3.**
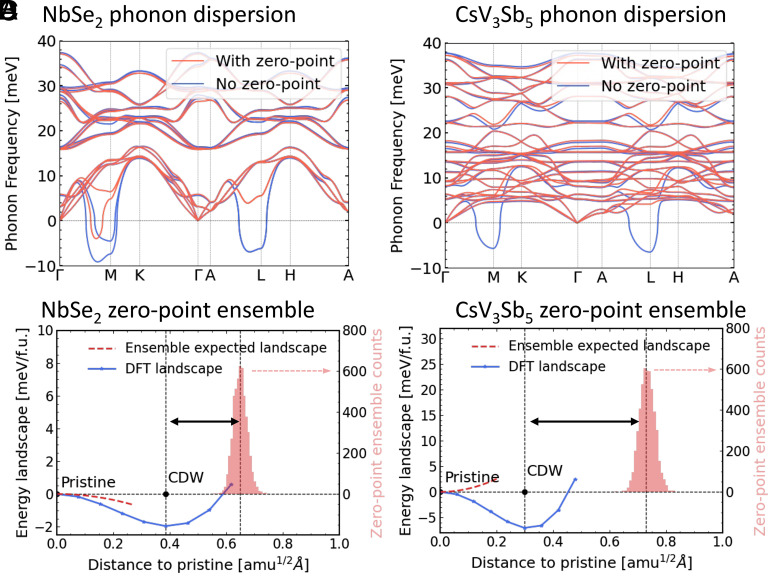
(*A*) The phonon dispersion of the pristine 2H-NbSe2 structure calculated without zero-point motion (“No zero-point”) by finite-displacement methods and with zero-point motion at 5 K (“With zero-point”) by SSCHA. (*B*) Similar to panel (*A*) but for CsV_3_Sb_5_. The stabilized phonon at 5 K suggests that the CsV_3_Sb_5_ pristine structure does not exhibit phonon softening behavior due to zero-point motion. (*C*) The blue dotted line depicts the DFT energy landscape of 2H-NbSe2 (*Left* y-axis, energy per formula unit). The horizontal axis “distance to pristine,” defined as $\sqrt{\sum_{i}M_{i}{\left(R_{i}-r_{i}\right)}^{2}/N}$, indicates the deviation of structures from the pristine cell. The *Right* y-axis illustrates the distribution of 5,000 structures in the zero-point ensemble. The expected free energy landscape for sampling this ensemble is sketched by a red dashed line, showing a smeared negative parabola. (*D*) Similar to panel (*C*) but for CsV_3_Sb_5_. The red dashed line reveals a positive parabola because the CsV_3_Sb_5_ CDW potential well is not sufficiently broad to be captured by the ensemble.

The analysis of phonon softening in 1H-NbSe2 and 2H-NbSe2 including both thermal and zero-point fluctuations by SSCHA has been performed before (61). Here, we first replicated the phonon dispersion calculations for 2H-NbSe2, as shown in Fig. 3*A*. The blue curve represents the phonon dispersion without considering zero-point motion (“No zero-point”) calculated using the finite displacement method and its displacement amplitude was set as 0.01 Å. In contrast, the red curve illustrates the phonon dispersion with zero-point motion at 5 K (“With zero-point”), performed via SSCHA. For the 5 K calculation with zero-point motion, thermal effects are negligible, yielding results equivalent to those at 0 K, and we selected 5 K to avoid potential numerical divergence issues. Our results were consistent with the previous study using SSCHA (61), where the imaginary frequency of “With zero-point” is around −5 meV near M point, validating our approach and computational setup.

Next, we applied the same procedure to CsV_3_Sb_5_. Surprisingly, the 5 K “With zero-point” phonon dispersion exhibited dynamic stability without imaginary phonon frequencies, as shown in Fig. 3*B*, which is in sharp contrast to the case in 2H-NbSe2. Fig. 3*B* differs from another SSCHA study on CsV_3_Sb_5_ (31), which we believe is most likely due to the use of a different supercell size. To understand the difference between 2H-NbSe2 and CsV_3_Sb_5_, we examined the optimized ensembles used for computing the free-energy Hessian matrices and observed significant differences in the distributions of the 5,000 ensemble configurations used in both calculations. As depicted in Fig. 3 *C* and *D*, the blue dotted line represents the energy landscape along the CDW distortion from the pristine structure calculated using DFT, with energy per formula unit referenced to the pristine structure. The CDW landscape is chosen to represent the most stable CDW structure for CsV_3_Sb_5_ and 2H-NbSe2, respectively [See *SI Appendix* for the 2H-NbSe2 CDW structure]. The horizontal axis, “distance to pristine,” is defined as the averaged mass-weighted distance to the pristine structure $\sqrt{\sum_{i}M_{i}{\left(R_{i}-r_{i}\right)}^{2}/N}$, where N is the number of atoms in a given structure, $M_{i}$ is the mass of the i-th atom in atomic mass units (amu), $R_{i}$ is the Cartesian coordinate of the i-th atom in the given distorted structure, $r_{i}$ is the Cartesian coordinate of the corresponding atom in the pristine cell. This value serves as a one-dimensional representation of the multidimensional atomic displacements, quantifying the normalized radial deviation of a given structure from its pristine state. The right y-axis illustrates the distribution of zero-point ensemble configurations based on their radial distances to the pristine structure. *SI Appendix*, section IB provides a simplified model to derive this distribution, which shows a Gaussian-like profile centered around the effective position uncertainty from quantum zero-point motion.

The “No zero-point” phonon dispersion, calculated by 0.01 Å finite-displacement method, probes the immediate vicinity of the pristine cell within the DFT landscape. In contrast, the ensemble due to zero-point motion detects a broader range of distances extending beyond the CDW potential well in Fig. 3 *C* and *D*. The first vertical dashed line of each figure denotes the location of the CDW potential well, whereas the second vertical dashed line indicates the average distance of configurations from the pristine structure in the optimized zero-point ensemble. The black double arrow represents the distance between these two vertical dashed lines and is almost twice larger in CsV_3_Sb_5_ compared to that in 2H-NbSe2. The red dashed lines denote the expected free-energy landscapes as a result of the phonon dispersions sampling from the zero-point ensemble. The red dashed line in Fig. 3*C* reveals an expected landscape with a smeared negative parabola for pristine 2H-NbSe2, suggesting that the zero-point ensemble effectively samples the CDW potential well. In other words, even though the expected free energy landscape in 2H-NbSe2 is smeared by the position uncertainty due to zero-point motions, the pristine structure remains dynamically unstable with imaginary phonon modes. In contrast, Fig. 3*D* reveals an expected landscape with a positive parabola for CsV_3_Sb_5_, since the average distance from the pristine structure of the zero-point ensemble is much wider than the CDW potential well. In this case, the CDW potential well cannot be effectively sampled by the ensemble, resulting in stabilized phonons of the pristine structure even near zero temperature. As a result, the uncertainty of the atomic position introduced by zero-point motion effectively stabilizes the CsV_3_Sb_5_ phonons in the pristine structure. Near the CDW transition temperature (94 K), thermal fluctuations further broaden the ensemble sampling space and, together with quantum fluctuations, further stabilize the pristine structure, leading to the absence of phonon softening.

To further investigate why the absence of phonon softening is observed specifically in CsV_3_Sb_5_, we examine the relationship between the zero-point ensemble and the CDW potential well. From the simplified model in *SI Appendix*, the average distance of the zero-point distribution can be estimated using the quantum uncertainty relation, approximately $\sqrt{\frac{\hbar }{2\omega }}$, where ω represents the effective frequency of phonon modes. Thus, we can assess the quantum fluctuations in the pristine phases from phonon dispersions. Given that the phonon dispersions in 2H-NbSe2 and CsV_3_Sb_5_ are similar, the zero-point ensemble average distances are comparable: 0.65 ${\mathrm{amu}}^{1/2}$Å for 2H-NbSe2 and 0.73 ${\mathrm{amu}}^{1/2}$Å for CsV_3_Sb_5_. What distinguishes these two cases is the relative distances of the CDW structures to the pristine structures. In 2H-NbSe2, the CDW structure is located at a distance 0.39 ${\mathrm{amu}}^{1/2}$Å from pristine, while in CsV_3_Sb_5_, this distance is shorter, at 0.30 ${\mathrm{amu}}^{1/2}$Å. This indicates that the distorted CDW structure in CsV_3_Sb_5_ is relatively closer to the pristine structure than 2H-NbSe2, which plays a crucial role in stabilizing the phonons.

Other than CsV_3_Sb_5_, it has been shown that in SrTiO3 the ferroelectric order is suppressed by quantum fluctuations (37, 38), and in 2H-NbSe2 quantum fluctuations can suppress the CDW phase transition at elevated pressures (62). These examples suggest that quantum fluctuations may play a significant role in materials containing relatively heavy elements. For instance, the paraelectric ground state of SrTiO_3_ stabilized by quantum fluctuations has been studied in detail with SSCHA (38). Although the ferroelectric phase of SrTiO_3_ has a lower energy, the tetragonal paraelectric phase remains dynamically stable down to 0 K due to quantum fluctuations, preventing the ferroelectric transverse optical (TO) phonon mode at the zone-center from softening and becoming unstable (38) [although partial softening of the TA phonon near Γ was recently observed, which was attributed to the development of mesoscopic ferroelectric domains (63)]. Extrapolating the temperature-dependent frequency of the ferroelectric TO mode in SrTiO_3_ suggests that the free energies of the paraelectric phase and the ferroelectric phase cross-over around 35 K, much lower than that in CsV_3_Sb_5_ (94 K). It is possible that at a much lower temperature, thermal and quantum fluctuations are not sufficiently strong to overcome the potential barrier separating the paraelectric and ferroelectric phases in SrTiO_3_, preventing a first-order transition. However, there are also experimental claims of the existence of ferroelectric domains in paraelectric SrTiO_3_ below roughly 40 K (63–65), which is analogous to the coexistence of pristine and CDW phases in CsV_3_Sb_5_. Due to the lower temperature and smaller energy scale in SrTiO_3_, this phase coexistence may be more sensitive to extrinsic factors, such as defects and strain. Our finding in CsV_3_Sb_5_ provides an alternative view to the prevailing understanding that phonon softening behavior is a necessary feature for EPC-induced CDW transitions. Instead, the extent of phonon softening depends on the competition between quantum fluctuations and the energy landscape that drives the transition and should lie in a continuous spectrum. For example, certain cuprates exhibit approximately 15% softening across the CDW transition (66, 67) and SrTiO_3_ shows 10 to 40% softening of its TA phonons at 20 K (63). Using our notion described above, cuprates and SrTiO_3_ are likely to display an intermediate zero-point ensemble distribution between the ones of complete-softening 2H-NbSe_2_ and no-softening CsV_3_Sb_5_.

### 2.3. Experimental Implications.

The lack of phonon softening in CsV_3_Sb_5_ also presents a significant deviation from the typical second-order phase transitions seen in conventional CDW materials. The phase transition in CDW materials is typically characterized by complete phonon softening, implying a continuous second-order transition. The softening behavior indicates that the curvature of the free energy landscape profile at the pristine structure, which reflects the phonon stability, decreases with decreasing temperatures. In the framework of Landau theory, this behavior indicates the continuous evolution of the pristine structure in the free energy landscape from a local minimum to a local maximum, implying a gradual modification of the order parameter from the pristine to the CDW phase.

In stark contrast, CsV_3_Sb_5_ exhibits a weak first-order phase transition, as evidenced by previous experiments, such as NMR (68, 69), X-ray scattering (70), heat capacity measurements (8), and thermal hysteresis behavior (71), etc. This behavior has previously been attributed to symmetry arguments, which allow for the presence of third-order terms in Landau theory (32). Our findings provide a physical explanation for the local stability of the pristine phase, which is stabilized by zero-point motion. As discussed in the previous section, we identified the dynamic stability of pristine CsV_3_Sb_5_ even below the CDW transition temperature. The stable pristine phase consistently represents an effective local minimum in the free energy landscape sketched in Fig. 2 *A* and *C*. The transition to the CDW phase is not accompanied by the gradual softening behavior. Instead, it occurs via a sudden shift in the order parameter indicated by the red arrow in Fig. 2*B*, characteristic of a first-order phase transition.

Additionally, the influence of quantum fluctuations implies that the first-order phase transition in CsV_3_Sb_5_ is likely weak. The zero-point ensemble samples a broad range of distances, leading to an effective potential landscape that is considerably smeared. Thus, the prominence of DFT energy barriers is reduced, further supporting the notion of a weak first-order transition. An immediate implication of CDW being the first-order transition is that the pristine and CDW phases coexist as distinct local minima near the transition temperature, a phenomenon also detected in CPS with a long-lifetime metastable state (49) and NMR with a narrow coexistence temperature range 91 K to 94 K (72). Another possible implication essentially suggests that the reduced energy barriers can be dynamically overcome, and thus we anticipate smearing effects in the X-ray diffraction measurements in CsV_3_Sb_5_ (73).

**Fig. 4.**
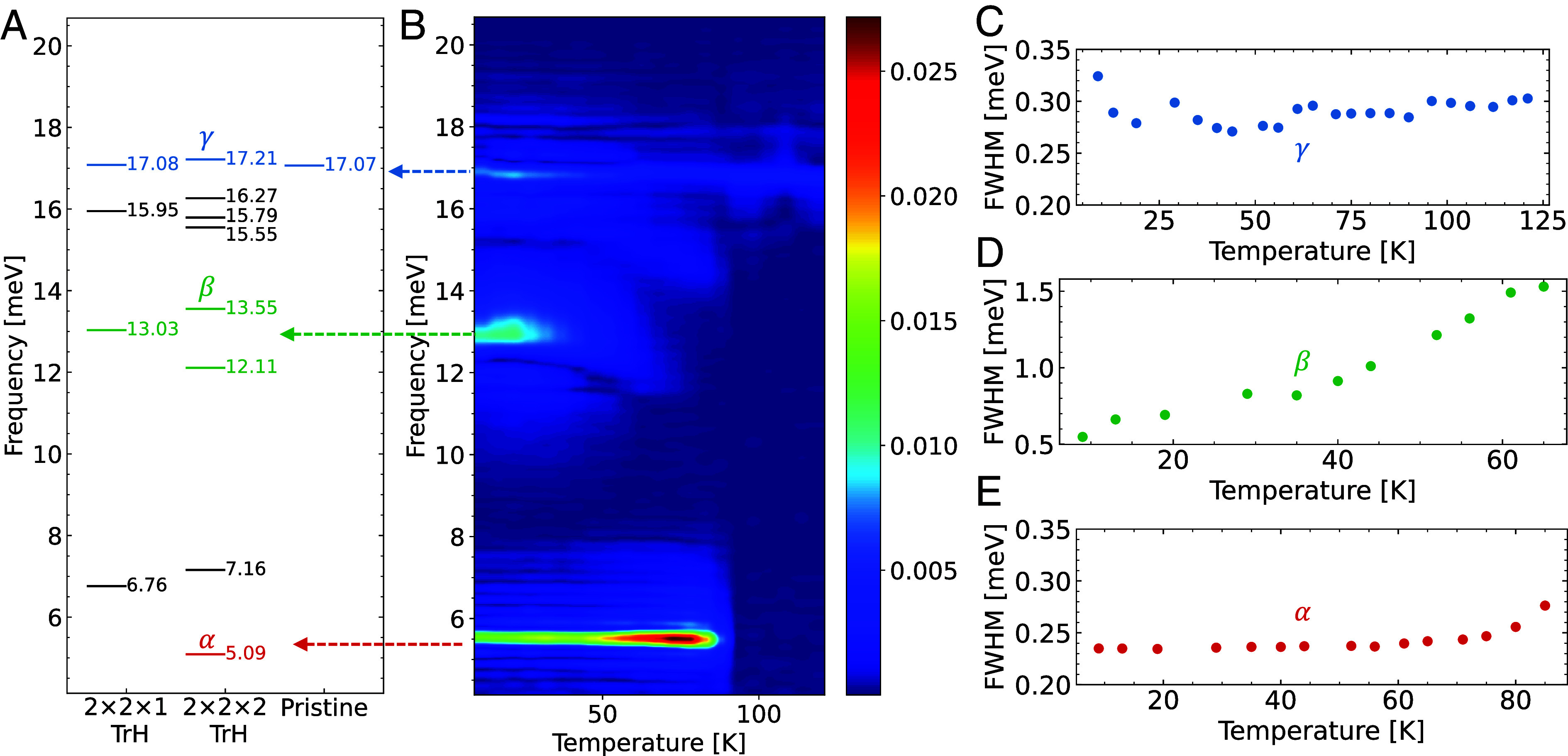
(*A*) Fully symmetric phonon modes at Γ point for the pristine 2×2×1 and 2×2×2 TrH orders. (*B*) Coherent phonon spectroscopy for CsV_3_Sb_5_. The evolution of full-width half maximum (FWHM) with temperatures for the (*C*) γ, (*D*) β, and (*E*) α modes.

We have also attempted to match the computational results with our CPS measurement of CsV_3_Sb_5_. Since CPS only observes the fully symmetric phonon mode, Fig. 4*A* includes the fully symmetric phonon modes at the Γ point of phonon dispersion for the CsV_3_Sb_5_ pristine, 2×2×1 TrH, and 2×2×2 TrH orders [see *SI Appendix* for the irreducible representations of Γ optical modes for 2×2×1 and 2×2×2 TrH orders]. The blue, green, and red colors are the γ,β,α modes aligned with the CPS result. These modes have been previously reported in ref. 49 from the M and L point of the pristine phonon dispersion.

The γ mode at 17.0 meV(4.1 THz), present at all temperatures, corresponds to the out-of-plane motion of the Sb atoms within the unit cell, oscillating toward and away from the Kagome plane (49). The α and β modes are induced by symmetry breaking as the system enters the CDW phases. The α mode at 5.5 meV(1.3 THz) emerges at 94 K and corresponds to the 5.09 meV(1.23 THz) mode in the 2×2×2 TrH structure, supporting the transition picture described in Fig. 2*B*, where the 2×2×2 TrH structure is the most stable phase that first occurs at the transition temperature. The β mode at 13.0 meV (3.1 THz) is broader and is observed only at lower temperatures around 60 K. This mode likely arises from the competition between different CDW structures. Specifically, the 2×2×1 TrH structure has a mode at 13.03 meV (3.15 THz), while the 2×2×2 TrH structure has modes at 13.55 meV and 12.11 meV (3.28 THz and 2.93 THz). This suggests that the metastable CDW structures, possibly including the 2×2×4 structures, have a set of modes with similar frequencies collectively leading to a broad β spectrum. The appearance of this β mode at 60 K signals the onset of metastable states, reflecting the coexistence of multiple CDW minima, as depicted in Fig. 2*C*. Our analysis has also identified additional modes in black that are active in CPS due to CDW distortions. The correlation of these newly identified peaks with the experimental observations is notably strong.

The typical effect of decreasing temperature on the CPS active modes is a narrowing of the peak linewidths due to reduced electron–phonon and phonon–phonon scatterings. However, as depicted in Fig. 4*C*–*E*, a saturation trend for the full-width at half maximum (FWHM) linewidth of the γ,β,α peaks emerges at lower temperatures [See *SI Appendix* for peak positions and peak areas of the γ,β,α modes]. Our theory offers a potential explanation for this saturation behavior. Below the CDW transition temperature ($T<T_{\mathrm{CDW}}$), as illustrated in Fig. 2*C*, the free energy profile introduces additional CDW orders as local minima with smeared energy barriers. These local minima could act as a source of disorder through competing CDW phases, which would contribute to the broadening of phonon peak linewidths, counteracting the narrowing effect of a lowering temperature. We propose that both the broadening and narrowing effects contribute to the observed saturation in the FWHM linewidth. This analysis elucidates the dynamical evolution of structural orders under thermal and zero-point effects, which is of fundamental importance in studying phase transitions in CDW materials.

## 3. Conclusion

In this study, we investigate the temperature-dependent free energy landscape associated with CDW structures in CsV_3_Sb_5_ using ab initio simulations, highlighting the important role of quantum zero-point motion. We identify an energy hierarchy of CDW structures including two chiral 2×2×4 structures, with the 2×2×2 TrH order being the most energetically favorable. Unlike typical CDW materials such as 2H-NbSe2, the inclusion of zero-point motion results in the stabilization of phonons in pristine CsV_3_Sb_5_ even below the transition temperature. In addition, quantum fluctuations can also smear the energy barriers, suggesting that CsV_3_Sb_5_ undergoes a weak first-order phase transition, accompanied by a coexistence of pristine and CDW phases near the transition temperature. We propose a dynamic evolution of the free energy landscape, in which certain stable higher-energy CDW states, such as the 2×2×1 TrH order, could occur at lower temperatures. Our theory aligns with CPS results, particularly in the additional local minima states contributing to the saturating linewidth trend of the γ, β, and α modes. Overall, this work challenges the conventional understanding that phonon softening must accompany EPC-induced CDW transitions. CsV_3_Sb_5_ exemplifies how quantum mechanical effects can significantly influence macroscopic properties and the nature of phase transitions. Given the complexity with competing energy landscape and quantum/thermal fluctuations, we expect that more experimental and computational data, especially finite-temperature simulations near the CDW transition temperature, can further clarify the physical picture sketched here. This research provides a unique perspective for further studies into other unconventional CDW materials and has rich implications in the interpretation of solid-state phase transitions.

## Supplementary Material

Appendix 01 (PDF)

## Data Availability

All study data are included in the article and/or *SI Appendix*.
