# Supplementary material for: Absence of phonon softening across a charge density wave transition due to quantum fluctuations
Source: Proc Natl Acad Sci U S A. 2025 Aug 1;122(31):e2507135122. doi: 10.1073/pnas.2507135122 (PMC12337337; doi:10.1073/pnas.2507135122)
Supplement: Supplementary file 1 — Appendix 01 (PDF) [file pnas.2507135122.sapp.pdf]

Supplementary Information: Absence of Phonon Softening across a Charge Density Wave Transition due to Quantum Fluctuations

Yubi Chen,[1,2] Terawit Kongruengkit,[3] Andrea Capa Salinas,[3] Runqing Yang,[2] Yujie Quan,[2] Fanghao Zhang,[2] Ganesh Pokharel,[3,4] Linus Kautzsch,[3] Stephen D. Wilson,[3] Sai Mu,[5,*] John W. Harter,[3,†] and Bolin Liao[2,‡]

[1]*Department of Physics, University of California, Santa Barbara, California 93106-9530, USA*

[2]*Department of Mechanical Engineering, University of California, Santa Barbara, CA 93106-5070, USA*

[3]*Materials Department, University of California, Santa Barbara, California 93106-5050, USA*

[4]*Perry College of Mathematics, Computing, and Sciences, University of West Georgia, Carrollton, GA 30118*

[5]*SmartState Center for Experimental Nanoscale Physics, Department of Physics and Astronomy, University of South Carolina, SC 29208, USA*

* mus@mailbox.sc.edu

† harter@ucsb.edu

‡ bliao@ucsb.edu

I. COMPUTATIONAL DETAILS

A. SSCHA method

The Stochastic Self-Consistent Harmonic Approximation (SSCHA) offers a robust quantum variational framework to study the anharmonic properties of materials at finite temperatures [1]. In the term "SSCHA", "Harmonic" refers to the utilization of a Gaussian form of the density matrix ρ, parameterized by the average atomic positions R and effective force constants Φ. The effective force constants, which also correspond to the variance in the Gaussian distribution, captures both quantum and thermal fluctuations. SSCHA aims to minimize the free energy $F[\rho]$, a functional of the Gaussian density matrix. This minimization of free energy F is achieved self-consistently by optimizing the density matrix ρ through variables R and Φ,

$$F_{\text{true}} \leq F[\rho] = E[\rho] - TS[\rho] \tag{1}$$

where F_{true} is the true free energy, $E[\rho]$ is the total energy, T is temperature, and $S[\rho]$ is the entropy.

The minimization gradient is derived from the ensemble average over a set of configurations sampled from the Gaussian distribution. The term "Stochastic" of SSCHA comes from the Monte Carlo nature of the distribution sampling. The phonon dispersion at finite temperatures is determined by diagonalizing the Hessian matrix of the free energy, expressed as $\frac{1}{\sqrt{m_a m_b}} \frac{\partial^2 F}{\partial R_a \partial R_b}$, where a, b are Cartesian indices, and m_a, m_b are the atomic masses. This method allows for an accurate representation of phonon dynamics including both thermal and quantum fluctuation effects.

B. Zero-point ensemble distribution

We can use a simplified model to estimate the distribution of the zero-point ensemble. The lattice Hamiltonian can be transformed into a set of independent harmonic oscillators, with each oscillator representing a phonon eigenmode characterized by a displacement amplitude x_i ($i = 1, \cdots, N$). These displacement amplitudes follow independent Gaussian distributions:

$$x_i \sim \exp\left(-\frac{x_i^2}{2\sigma^2}\right) \tag{2}$$

where the variance $\sigma^2 = \frac{\hbar}{m\omega_i}\left(\frac{1}{2} + \frac{1}{\exp\left(\hbar\omega_i/k_B T\right)-1}\right)$ is related to the phonon frequency ω_i through a finite-temperature expression of the position uncertainty, which reduces to $\frac{\hbar}{2m\omega_i}$ at zero temperature $T = 0$. Mass m can be absorbed into the definition of displacement x_i to eliminate the occurrence of m in the expression of σ^2. For simplicity, we assume a uniform variance σ^2, corresponding to an effective phonon frequency ω across all modes at temperature T. We define the variable r as the radial distance from the pristine structure (referred to as "distance to pristine" in the main text), given by $r = \sqrt{\sum_i x_i^2}$. This expression is invariant under a change of basis, allowing x_i to be atomic coordinates. The probability distribution for r becomes:

$$\exp\left(-\frac{x_1^2}{2\sigma^2}\right)\cdots\exp\left(-\frac{x_N^2}{2\sigma^2}\right)dx_1\cdots dx_N = C\exp\left(-\frac{r^2}{2\sigma^2}\right)r^{N-1}dr \tag{3}$$

where the angular coordinates have been integrated out, and C is a normalization constant. Thus, the distribution function is given by:

$$D(r) = C\exp\left(-\frac{r^2}{2\sigma^2}\right)r^{N-1} \tag{4}$$

To determine the average distance of the distribution $D(r)$, we evaluate:

$$\langle r^2\rangle = \frac{\int dr\exp\left(-\frac{r^2}{2\sigma^2}\right)r^{N+1}}{\int dr\exp\left(-\frac{r^2}{2\sigma^2}\right)r^{N-1}} = N\sigma^2 \tag{5}$$

This shows that the average distance of the zero-point ensemble is directly related to the position uncertainty σ, and therefore to the effective phonon frequency ω. In the main text, the factor N is eliminated as with the normalized definition of "distance to pristine" $r = \sqrt{\frac{1}{N}\sum_i x_i^2}$.

It is important to emphasize that our hypothesis, that "distance to pristine" is effective to describe the differences between the zero-point ensembles in 2H-$NbSe_2$ and CsV_3Sb_5, is a preliminary suggestion. We acknowledge that relying solely on "distance to pristine" might overlook information contained in the angular coordinates. This analysis of "distance to pristine" is a first step, and we hope that future studies will refine and establish a comprehensive criteria to characterize the effects of zero-point motion on CDW materials.

C. CsV_3Sb_5 convergence tests

Figure S1 illustrates the supercell convergence of the phonon dispersions for the pristine structure of CsV_3Sb_5. The blue curve corresponds to a 3×3×2 supercell (162 atoms) with

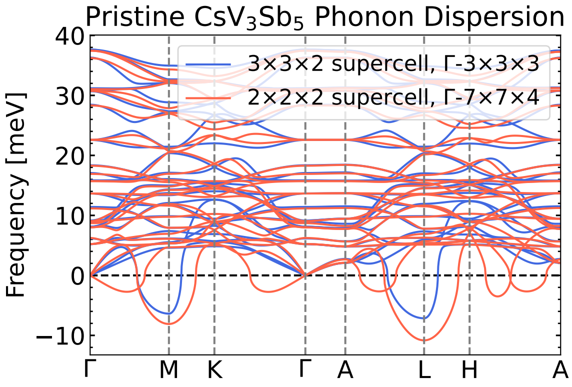

FIG. S1. The phonon dispersions for CsV_3Sb_5 pristine structure with different supercell sizes. The blue curve uses a 3×3×2 supercell (162 atoms) with a Γ-centered 3×3×3 **k**-point grid. The red curve uses a 2×2×2 supercell (72 atoms) and a Γ-centered 7×7×4 **k** grid.

a Γ-centered 3×3×3 **k**-point grid, while the red curve is obtained with a 2×2×2 supercell (72 atoms) and a Γ-centered 7×7×4 **k**-point grid. Based on the phonon dispersion analysis, we determined that a minimum supercell size of 3×3×2 with a Γ-centered 3×3×3 **k**-point grid is required to achieve convergent forces for the CsV_3Sb_5 pristine structures. Therefore, we used the consistent density functional theory (DFT) supercell size and **k** grid setups for the calculations of ensemble configurations in SSCHA.

In addition, the phonon dispersion convergence of CsV_3Sb_5 CDW structures requires a commensurate supercell. We have verified that the minimum requirement for 2×2×1 CDW and 2×2×2 CDW structures is a 288-atom supercell (2×2×2 stacking) with a Γ-centered 2×2×2 **k** grid, and for 2×2×4 CDW structures is a 576-atom supercell (2×2×1 stacking) with a 2×2×1 **k**-grid. Note the primitive cells of 2×2×2 TrH and 2×2×2 SoD structures both contain 36 atoms, which has the same number of atoms as 2×2×1 CDW structures.

Figure S2 displays the phonon dispersions for the pristine structure of CsV_3Sb_5, computed using different DFT functionals: Perdew–Burke–Ernzerhof with van der Waals correction (PBE+vdW), and PBE+vdW with spin-orbit coupling (PBE+vdW+SOC). Note that the pristine structure was relaxed only using PBE+vdW, with no subsequent relaxation performed after incorporating SOC with PBE+vdW. The similarity between the two phonon dispersion curves suggests that the inclusion of SOC has a minimal impact on the phonon dispersions and the atomic force constants.

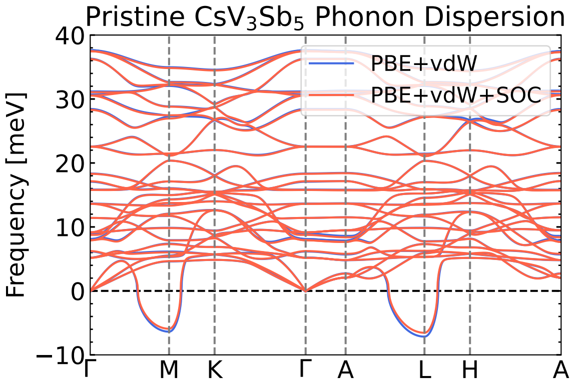

FIG. S2. The phonon dispersions for CsV_3Sb_5 pristine structure with PBE+vdW and PBE+vdW+SOC. Thus, SOC has negligible impact on phonon dispersions.

D. CsV_3Sb_5 CDW details

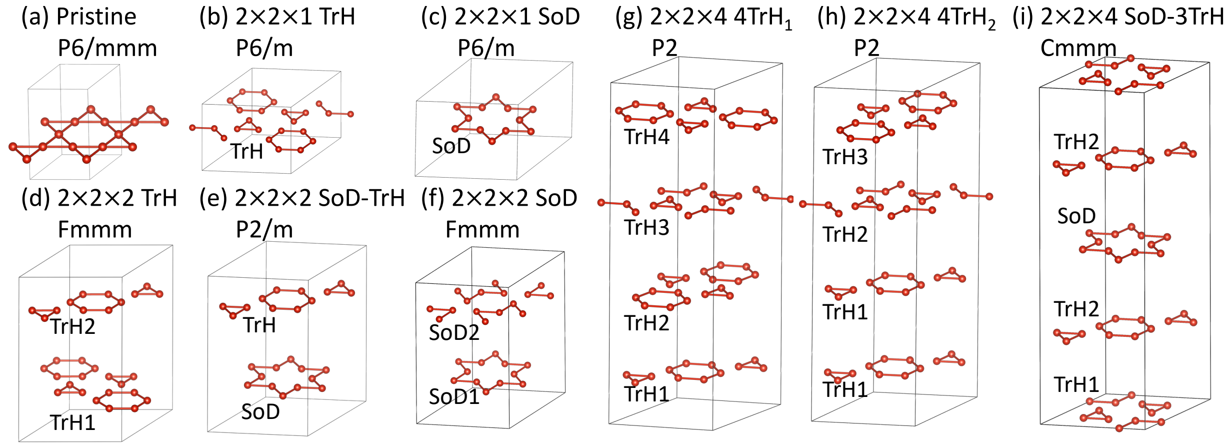

FIG. S3. CsV_3Sb_5 pristine and CDW structures

The computational setups of the PBE+vdW+SOC energies of CsV_3Sb_5 structures are set as below for a fair comparison. For structure relaxations, the **k** grids were consistently set to Γ-centered $12 \times 12 \times 4$ in the Brillouin zone of the primitive cell for structures with quadruple vertical folding ($2 \times 2 \times 4$ CDW orders), which means $6 \times 6 \times 1$ is the **k** grid used for the quadruple CDW structures. For the relaxation of smaller structures, a Γ-centered $18 \times 18 \times 6$ **k** grid is employed in the primitive cell Brillouin zone for those with less vertical folding ($2 \times 2 \times 1$ and $2 \times 2 \times 2$ CDW orders). The relaxation process utilized

the PBE+vdW functional. Since SOC has negligible effects on atomic forces, so we did not perform further relaxations the structures with PBE+vdW+SOC. We compared the energies of the structures after consistently stacking them into the $2 \times 2 \times 4$ supercells with 144 atoms, evaluated with PBE+vdW+SOC under a Γ-centered $6 \times 6 \times 1$ **k** grid (Γ-centered $12 \times 12 \times 4$ **k** grid in the pristine Brillouin zone).

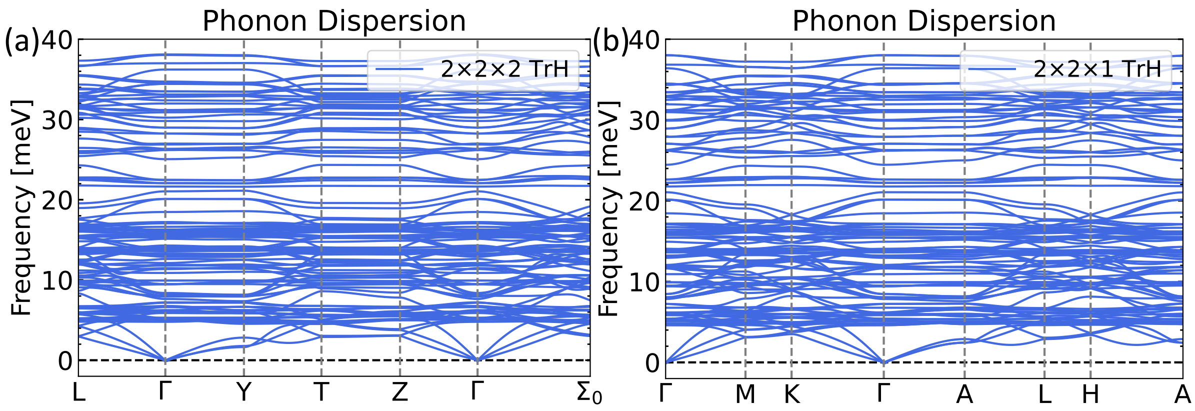

FIG. S4. The finite-displacement phonon dispersions of (a) 2×2×2 TrH structure and (b) 2×2×1 TrH structure.

Figure S3 contains the atomic structures of the identified CsV_3Sb_5 CDW orders. In the pristine structure, the V-V pairs are connected with the same bond length 2.725 Å. While in the CDW structures, the V-V bond distance cutoff is set as 2.7 Å,and pairs of V atoms that exceed this distance are not connected. Figure S3(a) is the pristine structure under symmetry P6/mmm, where V atoms form Kagome lattice. Figure S3(b) is the 2×2×1 trh-hexagonal (TrH) structure under symmetry P6/m, where the V atoms distorts into a TrH configuration. Figure S3(c) is the 2×2×1 Star-of-David (SoD) structure also under symmetry P6/m, where the V atoms distorts into a SoD configuration. Figure S3(d) is the 2×2×2 TrH structure under symmetry Fmmm, where the two V layers (TrH1 and TrH2) are horizontally shifted. Figure S3(e) is the 2×2×2 SoD-TrH structure under symmetry P2/m, where one V layer is TrH and another is SoD. Figure S3(f) is the 2×2×2 SoD structure under symmetry Fmmm, where the two V layers (SoD1 and SoD2) are horizontally shifted. Figure S3(g) is the 2×2×4 $4TrH_1$ structure under symmetry P2, where the four V layers (TrH1, TrH2, TrH3, and TrH4) are all relatively shifted with respect to each other.

Figure S3(h) is the 2×2×4 4TrH$_2$ structure under symmetry P2, where two V layers (TrH1) are the identical, but the other two V layers (TrH2 and TrH3) are shifted. Figure S3(i) is the 2×2×4 SoD-3TrH structure under symmetry Cmmm. This structure is reported in Ref. [2] containing one SoD layer and three TrH layers.

Figure S4 contains the phonon dispersions of 2×2×2 TrH and 2×2×1 TrH structures. Both phonon dispersions were calculated using $4 \times 4 \times 2$ supercells (288 atoms) and a Γ-centered $2 \times 2 \times 2$ **k** grid (a $8 \times 8 \times 4$ **k** grid in the primitive Brillouin zone). These calculations confirm the stability of both phonon structures, as indicated by the absence of imaginary frequencies. Our phonon dispersions resembles those reported in Ref.[3–5]. The high symmetry points are obtained from Vaspkit. [6]

E. NbSe$_2$ atomic structures

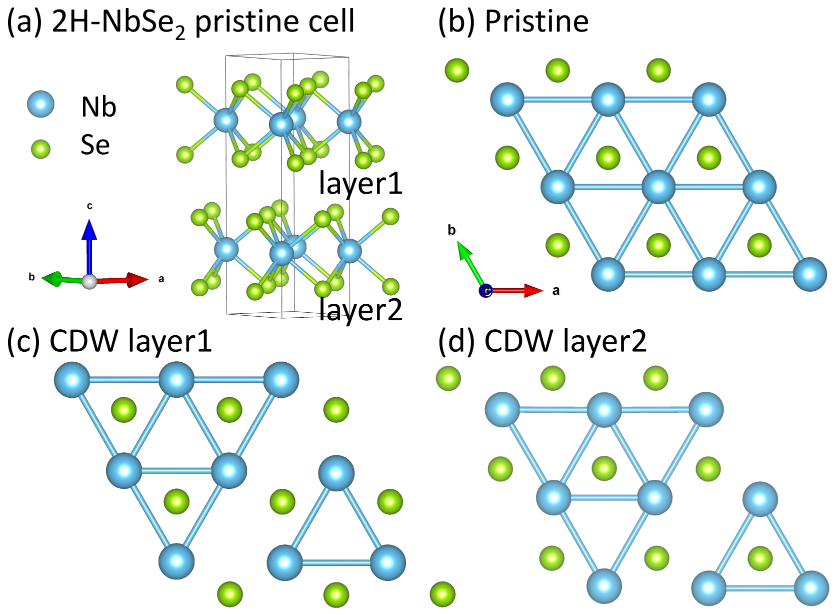

FIG. S5. (a) 2H-NbSe$_2$ pristine structure (b) Top view of the pristine structure (c) Top view of the layer 1 in the most stable CDW structures. (d) Top view of layer 2 in the CDW structure

Figure S5(a) shows the pristine cell of 2H-NbSe$_2$, and Fig. S5(b) provides a top view of the pritine cell. The most stable NbSe$_2$ CDW structure is obtained from Ref.[7–10], and its two layers are shown in Fig.S5(c,d). In this CDW structure, the Nb-Nb bond distance is set with a cutoff of 3.45 Å, and pairs of Nb atoms that exceed this distance are not connected.

II. COHERENT PHONON SPECTROSCOPY

A. Additional Coherent phonon spectroscopy data

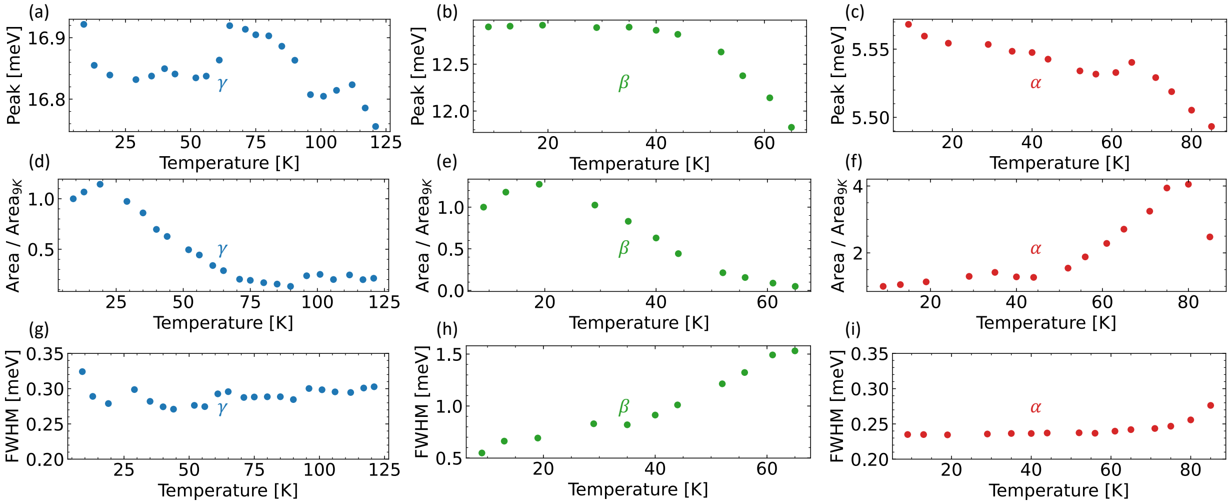

FIG. S6. Coherent phonon spectroscopy. (a,b,c) peak position of the γ, β, and α modes. (d,e,f) normalized Area of the γ, β, and α modes. (g,h,i) Full-width half maximum (FWHM) of the γ, β, and α active modes.

Coherent phonon spectroscopy (CPS) is a technique for observing only fully symmetric phonon mode. Figure S6 shows the peak position, normalized Area, and linewidth (Full-width half maximum) of the γ, β, and α active modes in CPS.

B. Optical modes of phonon dispersion at Γ

We have summarized two tables of the irreducible representations (IRREP) of optical phonon modes at Γ point of the two stable CDW phases shown in Fig. S4.

Table SI. The optical modes of the 2×2×1 TrH phonon dispersion in Fig. S4(b), calculated by Phonopy [11]. The frequency (Freq.) is in meV/THz units.

Freq.(meV/THz)	IRREP	Freq.(meV/THz)	IRREP	Freq.(meV/THz)	IRREP
4.61/1.11	E2u	12.94/3.13	Bu	22.63/5.47	E1u
4.85/1.17	Au	13.03/3.15	Ag	24.46/5.91	Ag
5.10/1.23	Bu	13.16/3.18	Bu	26.21/6.34	E2g

5.19/1.26	E1u	13.22/3.20	Bg	26.28/6.36	Bu
5.40/1.31	E1u	13.63/3.29	E1g	27.06/6.54	E1u
5.85/1.41	E1u	13.81/3.34	E2u	27.92/6.75	E2g
6.09/1.47	Bg	13.98/3.38	E1u	28.94/7.00	E2g
6.14/1.49	E1g	14.04/3.40	E1g	29.91/7.23	Ag
6.17/1.49	Au	14.93/3.61	Bg	30.01/7.26	Ag
6.18/1.50	Bu	15.45/3.73	E1u	30.97/7.49	Bu
6.76/1.63	Ag	15.45/3.73	E2g	31.19/7.54	Au
7.23/1.75	E2g	15.76/3.81	E2g	31.22/7.55	E1u
7.84/1.90	Bu	15.95/3.86	Ag	31.94/7.72	Bg
8.03/1.94	E2u	15.97/3.86	E2u	32.49/7.86	Bu
8.06/1.95	Au	16.16/3.91	Au	32.82/7.94	Au
8.46/2.05	E1u	16.42/3.97	Bu	32.98/7.97	E1g
9.42/2.28	E1g	16.74/4.05	Bu	33.44/8.09	E2u
9.50/2.30	Au	17.08/4.13	Ag	34.36/8.31	E1g
9.91/2.40	E2u	17.16/4.15	E1u	34.49/8.34	E1u
10.92/2.64	Au	18.45/4.46	Bg	36.41/8.80	Bu
11.63/2.81	E1u	20.15/4.87	E1u	36.85/8.91	Bg
11.82/2.86	E2u	21.06/5.09	Bu	38.01/9.19	E2u
11.94/2.89	Au	21.77/5.26	Ag		
12.05/2.91	E2g	22.22/5.37	E2g		

Table SII. The optical phonon modes at Γ point of the 2×2×2 TrH phonon dispersion in Fig. S4(a). The frequency (Freq.) is shown in meV/THz units.

Freq.(meV/THz)	IRREP	Freq.(meV/THz)	IRREP	Freq.(meV/THz)	IRREP
4.79/1.16	B2u	12.11/2.93	Ag	22.06/5.33	B2g
5.00/1.21	B3u	12.61/3.05	B2g	22.07/5.34	Ag
5.07/1.23	B2g	12.89/3.12	B1u	22.45/5.43	B3u
5.09/1.23	Ag	13.04/3.15	B3u	22.48/5.44	B1u
5.10/1.23	B3u	13.28/3.21	B1g	25.05/6.06	Ag
5.23/1.26	B1g	13.55/3.28	Ag	26.18/6.33	Ag
5.26/1.27	B1u	13.72/3.32	B1g	26.20/6.34	B1u

5.30/1.28	B3g	13.74/3.32	B3g	26.34/6.37	B2g
5.88/1.42	B3g	13.82/3.34	Au	26.88/6.50	B3u
5.94/1.44	B1g	13.83/3.34	B2u	26.99/6.53	B1u
6.07/1.47	B1u	13.85/3.35	B1u	28.24/6.83	B2g
6.21/1.50	B3g	13.94/3.37	B3u	28.25/6.83	Ag
6.24/1.51	B2u	14.13/3.42	B3g	28.99/7.01	B2g
6.62/1.60	B1g	14.29/3.46	B1g	28.99/7.01	Ag
6.63/1.60	B2g	15.13/3.66	B3g	29.81/7.21	B2g
6.76/1.63	B3g	15.30/3.70	B3u	30.23/7.31	Ag
7.16/1.73	Ag	15.35/3.71	B1u	30.89/7.47	B1u
7.21/1.74	B2g	15.55/3.76	Ag	31.14/7.53	B1u
7.63/1.84	B1u	15.57/3.76	B2g	31.17/7.54	B3u
7.94/1.92	Au	15.77/3.81	B2g	31.18/7.54	B2u
7.95/1.92	B2u	15.79/3.82	Ag	31.99/7.73	B3g
8.07/1.95	B2u	15.99/3.87	Au	32.38/7.83	B3u
8.30/2.01	B1u	15.99/3.87	B2u	32.82/7.94	B2u
8.38/2.03	B3u	16.16/3.91	B2u	33.07/8.00	B1g
9.46/2.29	B3g	16.27/3.93	Ag	33.11/8.01	B3g
9.47/2.29	B1g	16.28/3.94	B1u	33.49/8.10	B2u
9.49/2.30	Au	16.62/4.02	B3u	33.50/8.10	Au
9.92/2.40	B2u	17.06/4.12	B1u	34.43/8.33	B1u
9.94/2.40	Au	17.11/4.14	B3u	34.43/8.33	B3u
10.87/2.63	B2u	17.21/4.16	Ag	34.54/8.35	B1g
11.36/2.75	B3u	18.47/4.47	B3g	34.72/8.40	B3g
11.47/2.77	B1u	20.09/4.86	B3u	36.22/8.76	B3u
11.80/2.85	B2u	20.09/4.86	B1u	37.00/8.95	B1g
11.81/2.86	Au	21.04/5.09	B1u	38.03/9.20	Au
11.91/2.88	B2u	21.64/5.23	B2g	38.10/9.21	B2u

Supplementary References

---

[1] L. Monacelli, R. Bianco, M. Cherubini, M. Calandra, I. Errea, and F. Mauri, The stochastic self-consistent harmonic approximation: Calculating vibrational properties of materials with full quantum and anharmonic effects, J. Phys. Condens. Matter **33**, 363001 (2021).

[2] L. Kautzsch, B. R. Ortiz, K. Mallayya, J. Plumb, G. Pokharel, J. P. C. Ruff, Z. Islam, E.-A. Kim, R. Seshadri, and S. D. Wilson, Structural evolution of the kagome superconductors AV_3Sb_5 (A = K, Rb, and Cs) through charge density wave order, Phys. Rev. Materials **7**, 024806 (2023).

[3] G. Liu, X. Ma, K. He, Q. Li, H. Tan, Y. Liu, J. Xu, W. Tang, K. Watanabe, T. Taniguchi, L. Gao, Y. Dai, H.-H. Wen, B. Yan, and X. Xi, Observation of anomalous amplitude modes in the kagome metal CsV_3Sb_5, Nat. Commun. **13**, 3461 (2022).

[4] Q. Wang, P. Kong, W. Shi, C. Pei, C. Wen, L. Gao, Y. Zhao, Q. Yin, Y. Wu, G. Li, H. Lei, J. Li, Y. Chen, S. Yan, and Y. Qi, Charge density wave orders and enhanced superconductivity under pressure in the kagome metal CsV_3Sb_5, Adv. Mater. **33**, 2102813 (2021).

[5] A. Subedi, Hexagonal-to-base-centered-orthorhombic 4q charge density wave order in kagome metals KV_3Sb_5, RbV_3Sb_5, and CsV_3Sb_5, Phys. Rev. Materials **6**, 015001 (2022).

[6] V. Wang, N. Xu, J.-C. Liu, G. Tang, and W.-T. Geng, VASPKIT: A user-friendly interface facilitating high-throughput computing and analysis using VASP code, Comput. Phys. Commun. **267**, 108033 (2021).

[7] F. Zheng, Z. Zhou, X. Liu, and J. Feng, First-principles study of charge and magnetic ordering in monolayer $NbSe_2$, Phys. Rev. B **97**, 081101 (2018).

[8] J. A. Silva-Guillén, P. Ordejón, F. Guinea, and E. Canadell, Electronic structure of 2H-$NbSe_2$ single-layers in the CDW state, 2D Mater. **3**, 035028 (2016).

[9] B. Guster, C. Rubio-Verdú, R. Robles, J. Zaldívar, P. Dreher, M. Pruneda, J. A. Silva-Guillén, D.-J. Choi, J. I. Pascual, M. M. Ugeda, P. Ordejón, and E. Canadell, Coexistence of elastic modulations in the charge density wave state of 2H-$NbSe_2$, Nano Lett. **19**, 3027 (2019).

[10] A. Ptok, A. Kobiałka, M. Sternik, J. Łażewski, P. T. Jochym, A. M. Oleś, and P. Piekarz, Dynamical study of the origin of the charge density wave in AV_3Sb_5 (A= K, Rb, Cs) compounds, Phys. Rev. B **105**, 235134 (2022).

[11] A. Togo and I. Tanaka, First principles phonon calculations in materials science, Scr. Mater. **108**, 1 (2015), arXiv:1506.08498.
